# The role of the epidermis enhancer element in positive and negative transcriptional regulation of *ebony* in *Drosophila melanogaster*

**DOI:** 10.1093/g3journal/jkac010

**Published:** 2022-01-13

**Authors:** Noriyoshi Akiyama, Shoma Sato, Kentaro M Tanaka, Takaomi Sakai, Aya Takahashi

**Affiliations:** 1 Department of Biological Sciences, Tokyo Metropolitan University, Hachioji 192-0397, Japan; 2 Research Center for Genomics and Bioinformatics, Tokyo Metropolitan University, Hachioji 192-0397, Japan

**Keywords:** *ebony*, enhancer, silencer, *Drosophila*, abdominal midline

## Abstract

The spatiotemporal regulation of gene expression is essential to ensure robust phenotypic outcomes. Pigmentation patterns in *Drosophila* are determined by pigments biosynthesized in the developing epidermis and the *cis*-regulatory elements of the genes involved in this process are well-characterized. Here, we report that the known primary epidermal enhancer is dispensable for the transcriptional activation of *ebony* (involved in light-colored pigment synthesis) in the developing epidermis of *Drosophila melanogaster*. The evidence was obtained by introducing an approximately 1 kbp deletion at the primary epidermal enhancer by genome editing. The effect of the primary epidermal enhancer deletion on pigmentation and on the endogenous expression pattern of a *mCherry*-fused *ebony* allele was examined in the abdomen. The expression levels of the *mCherry*-fused *ebony* in the primary epidermal enhancer-deleted strains were slightly higher than that of the control strain, indicating that the sequences outside the primary epidermal enhancer have an ability to drive an expression of this gene in the epidermis. Interestingly, the primary epidermal enhancer deletion resulted in a derepression of this gene in the dorsal midline of the abdominal tergites, where dark pigmentation is present in the wild-type individuals. This indicated that the primary epidermal enhancer fragment contains a silencer. Furthermore, the endogenous expression pattern of *ebony* in the 2 additional strains with partially deleted primary epidermal enhancer revealed that the silencer resides within a 351-bp fragment in the 5' portion of the primary epidermal enhancer. These results demonstrated that deletion assays combined with reporter assays are highly effective in detecting the presence of positively and negatively regulating sequences within and outside the focal *cis*-regulatory elements.

## Introduction

The spatiotemporal regulation of gene expression during the development of organisms results in diverse phenotypes. The *cis*-regulatory elements (CREs) are strings of nucleotides that differentially modulate the transcription levels of specific genes typically in an allele-specific manner. The most common CREs, enhancers and silencers, are located within a certain distance from the transcription start sites of the target gene and contain binding sites for the transcription activators or repressors (Spitz and Furlong 2012; Long *et al.* 2016). While these CREs are generally unique to each expression unit (Arnone and Davidson 1997; Stern 2000; Wray *et al.* 2003; Prud’homme *et al.* 2007; Carroll 2008) various enhancers are reported to exhibit functional redundancy or to cooperatively define the expression site boundaries (Hong *et al.* 2008; Perry *et al.* 2009, 2010, 2011; Frankel *et al.* 2010; Bothma *et al.* 2015; El-Sherif and Levine 2016). Also, a number of enhancers with pleiotropic functions have been reported (Nagy *et al.* 2018; Preger-Ben Noon *et al.* 2018; Sabarís *et al.* 2019; Xin *et al.* 2020).

The CREs in genes involved in body pigmentation pattern have been well-characterized in *Drosophila* and multiple modular enhancers that activate transcription in different body regions have been documented in detail (reviewed in Massey and Wittkopp 2016; Rebeiz and Williams 2017). For example, the distinct CREs of *yellow* that activate transcription in bristles, wing and body, and abdomen have been identified (Geyer and Corces 1987; Martin *et al.* 1989; Wittkopp *et al.* 2002; Jeong *et al.* 2006; Roeske *et al.* 2018). However, a recent study revealed that many sequence fragments in the regulatory region of *yellow* exhibit redundant and cryptic enhancer activities, suggesting that *cis*-regulatory modules are not as distinct as described previously and more amenable to evolutionary changes (Kalay *et al.* 2019).

A complex architecture of *cis*-regulatory region has also been implicated from the within-species comparisons of *cis*-regulatory sequences of *ebony*, another gene involved in body pigmentation. Polymorphisms in *ebony*, which encodes an enzyme of the melanin biosynthesis pathway, is the major causative factor determining the body pigmentation intensity in *Drosophilamelanogaster* (Pool and Aquadro 2007; Takahashi *et al.* 2007; Rebeiz *et al.* 2009; Telonis-Scott *et al.* 2011). The sequence polymorphisms of the primary epidermis enhancer (priEE), which was identified to be located in the upstream intergenic region of *ebony*, were analyzed in detail. Some single-nucleotide polymorphisms (SNPs) first identified in the African populations affected the enhancer function but were not associated with body pigmentation intensity in the Japanese, European, North American, and Australian populations (Takahashi and Takano-Shimizu 2011; Telonis-Scott *et al.* 2011; Bastide *et al.* 2013; Dembeck *et al.* 2015; Miyagi *et al.* 2015; Telonis-Scott and Hoffmann 2018). Also, a priEE haplotype associated with light body color was identified in the Iriomote and Australian populations but not in the African populations (Telonis-Scott and Hoffmann 2018). Furthermore, there were no SNPs or indels in the priEE that showed complete association with the allele-specific expression levels in the developing epidermis in 20 strains sampled from the *D.melanogaster* Genetic Reference Panel including some strains with identical priEE sequences exhibiting differential allele-specific expression levels (Mackay *et al.* 2012; Miyagi *et al.* 2015). These analyses suggest the possibility of the presence of sequences outside the priEE region that regulate the expression level of *ebony* in the epidermis.

The priEE segment was the only segment within the approximately 10 kbp regulatory region (including an upstream intergenic region and intron 1) that drove the expression of the reporter gene in the epidermis (Rebeiz *et al.* 2009). However, the previous findings above have indicated that the sequence variation within the priEE was not sufficient to explain the wide range of expression level variation of this gene. Therefore, we hypothesized that similar to *yellow*, *ebony* has other enhancers in addition to the priEE, possibly located outside the known approximately 10 kbp regulatory region and may be taking part redundantly in activating transcription of this gene in the developing epidermis.

In comparison to the studies of enhancers, information on silencers is limited partly due to technical difficulties in detecting repressive activities by reporter assays. However, some studies conducting careful dissections of the *cis*-regulatory region of *ebony* have located some regions containing the silencers that repress transcription at the corresponding dark areas of the abdomen (Rebeiz *et al.* 2009; Johnson *et al.* 2015). In *D. melanogaster*, dark stripes are visible in the posterior regions of the A2–7 abdominal tergites in females and the A2–4 tergites in males. Also, the abdominal pigmentation pattern exhibits sexual dimorphism with totally dark A5–7 tergites observed only in males. Furthermore, another characteristic dark line is present along the dorsal midline of the abdominal tergites in both males and females of this species. The dark line is a characteristic of the subgenus *Sophophora* with some exceptions (Markow and O’Grady 2006) and the expression of *ebony* is not present in this region (Rebeiz *et al.* 2009; Hughes *et al.* 2020). The locations of silencers responsible for stripe repression and male-specific repression in the posterior tergites have been indicated but with a limited resolution (Rebeiz *et al.* 2009; Johnson *et al.* 2015). Moreover, no silencers that establish repression at the dorsal midline have been identified previously.

The transgenic reporter assay is a powerful approach to dissect regulatory sequences and identify CREs, such as enhancers and silencers. However, some limitations exist because this assay does not test the sequence fragments in their native genomic environment (Halfon 2019). Especially, the lengths and borders of the sequence fragments can markedly affect the results (Kalay *et al.* 2019). For the silencer screening, the test sequences need to be connected to or placed together with an enhancer in front of the reporter gene to test for negative regulations (Rebeiz *et al.* 2009; Johnson *et al.* 2015; Gisselbrecht *et al.* 2020).

In this study, rather than conducting reporter gene assays, we examined the genomic region by modifying the endogenous upstream sequence using the clustered regularly interspaced short palindromic repeats (CRISPR)-CRISPR associated protein 9 (Cas9) system. The deletion of the known priEE in a single genetic background enabled the examination of its contribution to the body color phenotype. We combined it with an assay using a reporter gene fused to the endogenous *ebony* to capture the changes in the expression pattern. As a result, we uncovered the presence of the enhancer activities outside the known priEE that drive the broad expression of *ebony* in the developing epidermis. We also show that the priEE fragment contains a silencer for repressing the expression of *ebony* in the dorsal midline of the abdominal tergites, which is necessary for developing the *Sophophora*-specific pigmentation pattern. We discuss the consequences of such regulatory system on the evolution of CREs and the potential application of a similar approach to other genomic regions.

## Materials and methods

### Fly strains

*y^2^* *cho^2^* *v^1^* *P{nos* *-Cas9*, *y^+^*, *v^+^}1A*/*FM7c*, *Kr*-*GAL4UAS*-*GFP* (Cas-0002), *y^1^v^1^P{nos-phiC31\int.NLS}X*; *attP40 (II)* (TBX-0002), *y^2^cho^2^v^1^*; *Sco*/*CyO* (TBX-0007), and *y^2^cho^2^v^1^*; *PrDr*/*TM6C*, *SbTb* (TBX-0010) lines were obtained from the NIG-FLY Stock Center. *w^1118^*; *wg^Sp-1^/CyO*; *Pr^1^ Dr^1^/TM3*, *Sb^1^ Ser^1^* (DGRC#109551) and *e*^1^ (DGRC#106436) were obtained from the Kyoto Stock Center. The isogenized Cas-0002 strain (Cas-0002-iso) was established via the triple balancer by crossing DGRC#109551 with Cas-0002 (Supplementary Fig. 1). The TBX-double-balancer (*y^2^cho^2^v^1^*; *Sco*/*CyO*; *PrDr*/*TM6C*, *SbTb*) was generated from TBX-0007 and TBX-0010. The 2 attP strains *y^1^ w^1118^*; *PBac{y^+^-attP-3B}VK00033* (DGRC#130419) and *y^1^ w^1118^*; *PBac{y^+^-attP-3B}VK00037* (DGRC#130421) were used to generate transgenes at WellGenetics. All fly stocks were reared at 25°C and maintained under a 12 h light: 12 h dark cycle on a standard corn-meal fly medium.

### Repair construct for *mCherry* knock-in

**Fig. 1.**
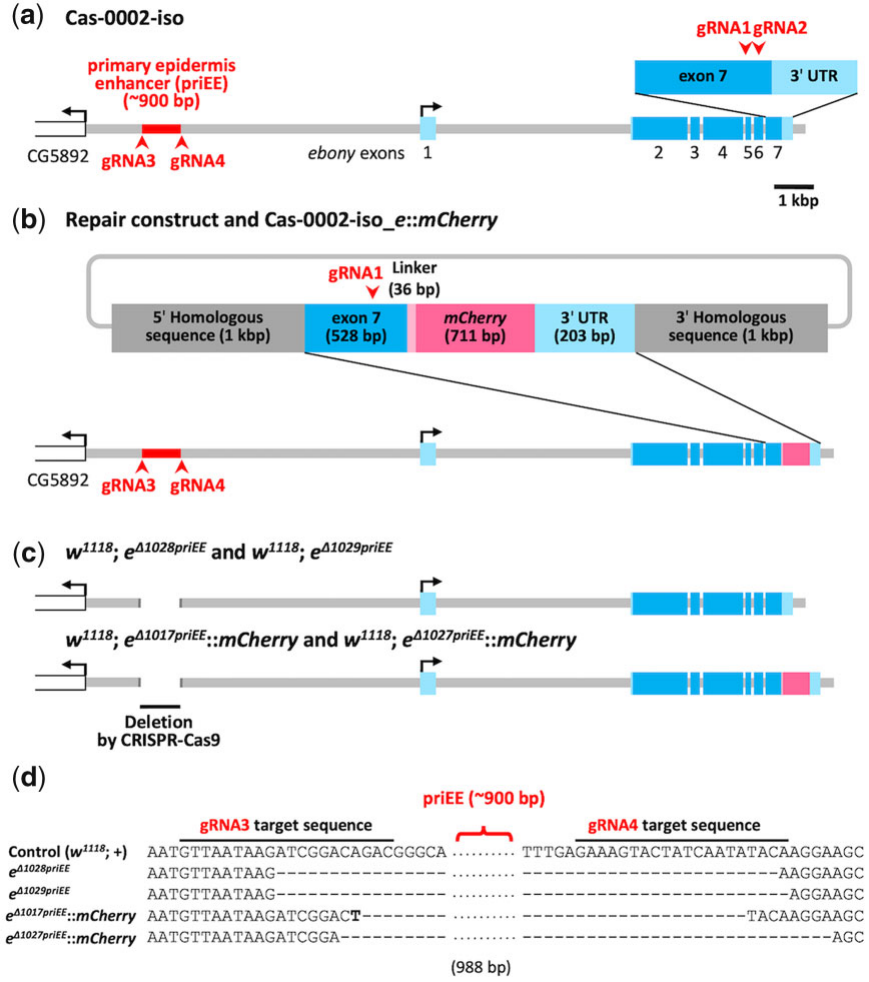
Construction of the primary epidermis enhancer (priEE) knockout strains. a) The genomic region surrounding *ebony* in Cas-0002-iso, an isogenic line carrying *nos*-*Cas9*. b) The genomic region surrounding *ebony* in Cas-0002-iso_*e*::*mCherry* is shown with the repair construct for *mCherry* knock-in. c) The genomic region surrounding *ebony* in strains with deleted priEE after the removal of *y^2^* (Supplementary Fig. 4). The light blue box indicates the untranlated region (UTR) and the blue box indicates the coding sequence (CDS). The red arrowhead indicates the target site of guide RNA (gRNA) sequences. d) Partial sequence alignment around the priEE fragment in the control and priEE-deleted strains. *w^1118^*; *e^Δ1017priEE^*::*mCherry* had a single T of unknown origin within the deleted region.

The repair construct (Fig. 1b) was designed following the method described by Hinaux *et al.* (2018). The construct *pJet-yellow_F4mut-mCherry* was gifted from Dr. Nicolas Gompel. A part of the *ebony* locus [2,610 bp (from approximately 1 kbp upstream of exon 7, to approximately 1 kbp downstream of 3'UTR)] was amplified from Cas-0002-iso (Fig. 1a) using primers with the XhoI, (5'-AGCctcgagTGGTGGATAAGGCCATTGTT-3') and XbaI (5'- CAGtctagaTGCAACTGGTTTGTGCGTAT-3') digestion sites. PCR was performed using KAPA HiFi HotStart ReadyMix (Kapa Biosystems). The *pJet-yellow_F4mut-mCherry* vector was digested with XhoI and XbaI and the fragments flanked by these digestion sites (including partial *yellow* and *mCherry* gene sequences) were replaced by the PCR product, which was digested with the same restriction enzymes. The complete sequence of the resulting vector, excluding the *ebony* termination codon, was PCR-amplified using the following primers: 5′-GACGACCACCCGGTGGACGT-3′ and 5′-TTTGCCCACCTCCTTCCAAT-3′.

Next, the *mCherry* sequence with a 5' linker (Waldo *et al.* 1999) was amplified from the *pJet-yellow_F4mut-mCherry* vector using primers with 15 bp homologous flanking sequences (5′-AAGGAGGTGGGCAAAGGATCCGCTGGCTCCGCTGCTG-3′ and 5′-CACCGGGTGGTCGTCTTACTTGTACAGCTCGTCCATGCC-3′). These 2 amplicons were fused using the In-Fusion HD Cloning Kit (TaKaRa) to generate the *pJet*-*ebony*-*mCherry* vector.

Finally, the 2 synonymous mutations were inserted at the target sequence of gRNA (5′-GCGCGCTATTGTCCATTGGA-3′) to reduce the risk of the repair construct being cut during the knock-in reaction. To induce mutations, 2 overlapping amplicons, including the gRNA target sequence, from the *pJet-ebony-mCherry* vector were generated using PCR with the following primer pairs: 5′-AATCCCCGCGAGAACATC-3′ and 5′-TCCAGTGTACAATAGCGCGC-3′; 5′-GCGCTATTGTACACTGGAAG-3′ and 5′-TTGTCTGGAAATCAAAGGCTTA-3′. These 2 PCR products were connected using overlap extension PCR to generate a mutated fragment. This mutated fragment was replaced by the original homologous sequence of the *pJet-ebony_mut-mCherry* vector by fusing the mutated fragment and the PCR product amplified from the *pJet-ebony-mCherry* vector using the In-Fusion HD Cloning Kit (TaKaRa) with the following primers: 5′-GCCTTTGATTTCCAGACAA-3′ and 5′-GTTCTCGCGGGGATTCAAC-3′. The constructed *pJet-ebony_mut-mCherry* vector was used as the repair construct for *mCherry* knock-in.

### gRNA vector cloning

All the guide sequences of gRNAs were cloned into the pCFD5 vector (Addgene ##73914) according to the pCFD5 cloning protocol (Port and Bullock 2016). The guide sequences of gRNA1 (5′-GGAGCACGAGGTTCTGCGGG-3′) and gRNA2 (5′-GCGCGCTATTGTCCATTGGA-3′) were designed within exon 7 of *ebony* and cloned into separate pCFD5 vectors. The guide sequences of gRNA3 (5′-GTTAATAAGATCGGACAGAC-3′) and gRNA4 (5′-GAAAGTACTATCAATATACA-3′), which were designed at both ends of the approximately 900-bp priEE fragment (Fig. 1d and Supplementary Fig. 2), were cloned into a single plasmid. Two additional guide sequences, gRNA5 (5′-TGAATAGTGATCAGCTGGTG-3′) and gRNA6 (5′-TATGAGCATCCATATATCAG-3′), were designed within the priEE fragment (Supplementary Fig. 3) and cloned into another single plasmid including gRNA3 and gRNA4. An In-Fusion HD Cloning Kit (TaKaRa) was used for cloning.

### Construct for reporter gene assay

The sequence of the priEE of *ebony* was amplified from Cas-0002-iso using the following primers with restriction enzyme digestion sites: 5'-CGGgaattcGGGCAAAGCAGGGTGAATA-3' (EcoRI site) and 5'-ACTgcggccgcTCACAGGGACTTATGGGAAA-3' (NotI site). These primers were designed to amplify most of the priEE knocked out sequences including the whole *e*_ECR0.9 (Takahashi and Takano-Shimizu 2011), *e*_core_*cis* (Miyagi *et al.* 2015), and “0.7 kb core abdominal element” (Rebeiz *et al.* 2009) (Supplementary Fig. 2). The amplified product and the pEGFP-attB vector with a minimal Hsp70 promoter (*Drosophila* Genomics Resource Center) were digested with EcoRI and NotI. The PCR product was cloned into the multi-cloning site of the vector.

### Embryonic microinjection

For *mCherry* knock-in, an *ebony* knockout strain was generated by injecting the gRNA1 guide-sequence-cloned pCFD5 vector (200 ng/μl) into the embryos of Cas-0002-iso. The embryos of *ebony* knockout strain were injected with a mixture of the gRNA2 guide-sequence-cloned pCFD5 vector (200 ng/μl) and the repair construct *pJet-ebony_mut-mCherry* (400 ng/μl). Of the 250 adult flies that emerged from the injected embryos, 4 restored wild-type body color. The sequences of exon 7 of *ebony* and knocked-in *mCherry* were confirmed using Sanger sequencing with a BrilliantDye Terminator cycle sequencing kit (NimaGen) and an ABI PRISM 3130xl Genetic Analyzer (Applied Biosystems). The established strain was named Cas-0002-iso_*e*::*mCherry* (Fig. 1b).

The pCFD5 vector with guide sequences of gRNA3 and gRNA4 (200 ng/μl) was injected into the embryos of TBX-0002. The gRNA expression strain (*y^2^cho^2^v^1^*; *attP40{gRNA, v^+^}*; *PrDr*/*TM6c*, *SbTb*) was established by mating the successfully transformed individual with the TBX-double-balancer. The guide sequences of gRNAs of the established strains were confirmed using Sanger sequencing with a BrilliantDye Terminator cycle sequencing kit (NimaGen) and an ABI PRISM 3130xl Genetic Analyzer (Applied Biosystems). To induce partial deletions of priEE, the embryos of *w^1118^*; *e*::*mCherry* were injected with a mixture of the gRNA3–6 cloned pCFD5 and pBFv-nosP-Cas9 vectors (200 ng/μl each) (Kondo and Ueda 2013).

The pEGFP-attB vector with the priEE fragment was prepared at a high concentration using Plasmid Midi Kit (Qiagen) and transported to WellGenetics (Taiwan) for injection into 2 attP strains (*y^1^ w^1118^*; *PBac{y^+^-attP-3B}VK00033* and *y^1^ w^1118^*; *PBac{y^+^-attP-3B}VK00037*).

### Deletion strains generated by CRISPR-Cas9

The deletion strains with breakpoints at gRNA3 and gRNA4 targeting sites were generated by crossing gRNA expression strains with Cas-0002-iso or Cas-0002-iso_*e*::*mCherry* (the crossing scheme shown in Supplementary Fig. 4). Deletions (Dels) occur in the germline cells of G1. Twelve G1 males were crossed one by one with several TBX-0010 virgin females. Eight G2 males sampled from the progenies of each G1 male were subjected to PCR screening. DNA samples extracted from the mid-legs of G2 males were amplified using the primers e_-5029F (5′-CGTGTGCCTGATCGCTAGA-3′) and e_-3391R (5′-ACTCGTGCCTTACTTAATCTGAA-3′), which were designed to amplify the target region. The G2 individuals were screened by subjecting the amplicons to electrophoresis using a 1% agarose gel. G2 individuals with deletions were crossed again with TBX-0010. Then, G3 (*y^2^cho^2^v^1^*; +; Del/*TM6c*, *SbTb*) individuals were crossed with each other to establish G4 homozygous strains (*y^2^cho^2^v^1^*; +; Del). The deletions were confirmed using Sanger sequencing with a BrilliantDye Terminator cycle sequencing kit (NimaGen) and an ABI PRISM 3130xl Genetic Analyzer (Applied Biosystems). The males from the homozygous deletion strains (G4) were crossed twice with the double balancer *w^1118^*; *wg^Sp-1^/CyO*; *Pr^1^Dr^1^/TM3, Sb^1^Ser^1^* (DGRC#109551) to replace the *y^2^cho^2^v^1^* X chromosome. Finally, the G7 homozygous deletion strains (*w^1118^*; +; Del) were established (Supplementary Fig. 4). Control strains (*w^1118^*; +; + and *w^1118^*; +; *e*::*mCherry*) were established with the same crosses using TBX-0002 instead of the gRNA expression strain (Supplementary Fig. 4).

The deletion strains with partial priEE deletion induced by the gRNA3–6 cloned vector were established by embryonic injection as described previously. The subsequent procedures were the same as above and the crosses were performed as in Supplementary Fig. 5.

### Quantification of pigmentation intensity

**Fig. 2:**
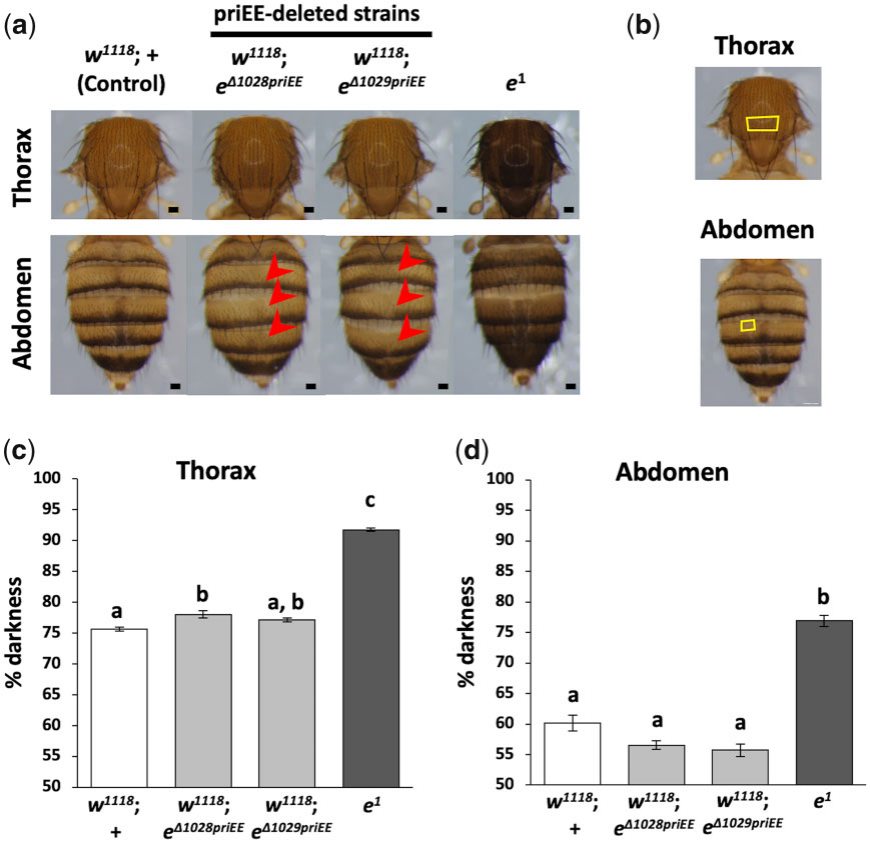
Effect of primary epidermis enhancer (priEE) knockout on the intensity and patterns of pigmentation. a) Images of the 5–7-day-old adult females. The red arrow indicates the area where the dark pigmentation in the dorsal midline is missing. b) Yellow squares indicating the areas of thorax (upper panel) and abdomen (lower panel) where pigmentation was quantified. c) Percent (%) darkness values obtained from the grayscale images of the thorax. d) Percent (%) darkness values obtained from the grayscale images of the A4 abdominal segment. *N* = 10 for each strain. Different letters indicate significant differences between strains (*P* < 0.05; Kruskal–Wallis rank sum test followed by Dunn’s test). Scale bars indicate 100 μm and error bars denote standard error.

At 5–7 days after eclosion, females were placed in 10% glycerol in ethanol at 4°C for 1 h. Next, the flies were rotated in 10% glycerol in phosphate-buffered saline (PBS) at room temperature for 1 h after removing the head, legs, and wings. The images of the dorsal body of flies soaked in 10% glycerol in PBS were captured using a digital camera (DP73, Olympus) connected to a stereoscopic microscope (SZX16, Olympus). The same parameters (exposure time, zoom width, and illumination) and reference grayscale (brightness = 128; ColorChecker, X-rite) were applied for capturing all images. White balance was corrected using the white scale (Brightness = 255; ColorChecker, X-rite) with cellSens Standard 1.6 software (Olympus). Pigmentation intensity was measured in manually selected areas of the thorax and abdomen (Fig. 2b) from RGB images of flies using ImageJ 1.53a (Schneider *et al.* 2012). The mode grayscale brightness values from thorax and abdomen were corrected using the reference grayscale of the background area at the bottom left corner of each image. The percent of darkness was calculated as follows:

$$\left(1-\frac{\mathrm{brightness}}{\mathrm{background}\;\mathrm{brightness}}\times \frac{128}{255}\;\right)\times 100\;(\%).$$

The raw measurement data are in Supplementary Table 1. The data were analyzed using the Kruskal–Wallis rank sum test followed by Dunn’s test. Statistical analyses were performed using R version 4.0.3 (R Core Team 2020).

### Confocal microscopy

The adult flies were dissected 4 h after eclosion and the abdomen, wings, front legs, and halteres were collected in PBS. The dorsal abdominal cuticle and epidermis were separated from the rest of the abdomen. The fat body, internal organs, and genitalia were gently removed. The head of adult females collected at 4–4.5 h after the light was turned on was dissected in PBS and the intact brain was obtained. Each brain sample was fixed in 4% paraformaldehyde for 1 h and washed with PBS for 1 h after fixation.

Each specimen was mounted with VECTASHIELD Mounting Medium with DAPI (Vector Laboratories) and imaged under a C2 plus confocal microscope (Nikon). Max intensity images were composited from the XY overlapping images (abdomen: 12 images, wing: 10 images) with 1 μm wide Z-stacks using the NIS Elements AR 4.50.00 software. The following laser wavelengths were applied for obtaining images: 488 nm activation wavelength and 509 nm imaging wavelength for EGFP imaging; 561 nm activation wavelength and 620 nm imaging wavelength for mCherry. The identical parameters of C2 plus settings (HV, offset, laser power, pinhole size, scan size, scan speed, scan direction, and zoom) were applied for imaging the same tissue (mCherry or EGFP). The images were taken under parameter settings that would not give saturated signals and no further corrections were applied.

### Quantification of mCherry fluorescent intensity

**Fig. 3:**
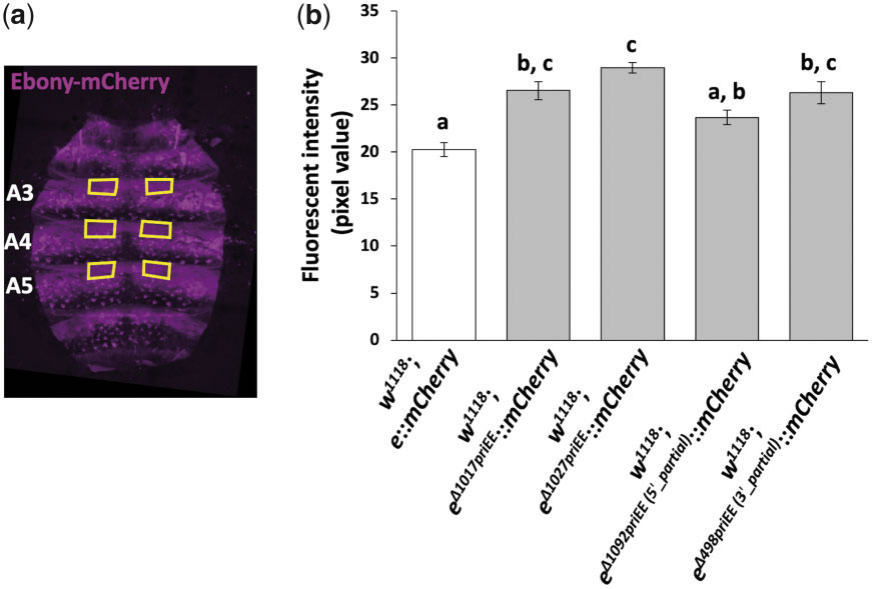
Effect of priEE knockout on the fluorescent intensity of mCherry fused to Ebony. a) Yellow squares indicating the areas of the abdominal tergites A3–5 where pigmentation was quantified in a confocal fluorescence image of the developing abdominal epidermis. b) Fluorescence intensities in pixel values (0–255) quantified from the images of the control (*w^1118^*; *e*::*mCherry*), the priEE-deleted strains (*w^1118^*; *e^Δ1017priEE^*::*mCherry* and *w^1118^*; *e^Δ1027priEE^*::*mCherry*), and the strains with partially deleted priEE (*w^1118^*; *e^Δ1092priEE(5'^***^*_*^**^*partial)*^::*mCherry* and *w^1118^*; *e^Δ498priEE(3'^***^*_*^**^*partial)*^::*mCherry*). *N* = 10 for each strain. Different letters indicate significant differences between strains (*P* < 0.05; one-way analysis of variance, followed by Tukey HSD post hoc test). Error bars denote standard error.

The fluorescent intensities of the confocal microscopic images of the abdomens from 10 individuals were quantified per strain. The mode pixel values (0–255) in the manually selected areas of the abdominal third, fourth and fifth segments (Fig. 3a) were measured by using ImageJ 1.53a (Schneider *et al.* 2012). The mode of the pixel values of the whole image, which reflects that of the dark background of each image was subtracted from the pixel values of the abdominal segments. The raw measurement data are in Supplementary Table 2. The data were analyzed using one-way analysis of variance (ANOVA), followed by Tukey HSD post hoc test. Statistical analyses were performed using R version 4.0.3 (R Core Team 2020).

## Results

The priEE fragment was precisely knocked out using the CRISPR-Cas9 system to examine whether transcriptional activation of *ebony* occurs in the absence of the priEE. First, to control the genomic background, an isogenic Cas-0002 line (Cas-0002-iso) carrying the *nos*-*Cas9* transgene was constructed (Fig. 1a and Supplementary Fig. 1). Next, *mCherry* was knocked-in to the 3' end of the *ebony* coding sequence in the Cas-0002-iso line using the CRISPR-Cas9 system (Fig. 1b). The resultant transgenic line (Cas-0002-iso_*e*::*mCherry*) was designed to produce a Ebony-mCherry fusion protein. Cas-0002-iso and Cas-0002-iso_*e*::*mCherry* lines were crossed with guide RNA (gRNA) expression lines to drive a targeted deletion at the approximately 900-bp priEE fragment (Takahashi and Takano-Shimizu 2011; Miyagi *et al.* 2015) (Supplementary Fig. 4). Additional crosses were performed to remove *y^2^* and replace the X chromosome with *w^1118^* to avoid interference from *yellow*, which is in the same pigment synthesis pathway (Supplementary Fig. 4). The following 4 priEE deletions were generated; 2 from Cas-0002-iso line (*w^1118^*; *e^Δ1028priEE^* and *w^1118^*; *e^Δ1029priEE^*) and 2 from Cas-0002-iso_*e*::*mCherry* line (*w^1118^*; *e^Δ1017priEE^*::*mCherry* and *w^1118^*; *e^Δ1027priEE^*::*mCherry*) (Fig. 1, c and d). If the priEE contains the only enhancer driving the expression of *ebony* in the epidermis, the priEE-deleted strains must exhibit a dark body color equivalent to the *ebony* null mutant (*e^1^*). Contrary to this prediction, the body color of the priEE-deleted strains was similar to that of the control strain (Fig. 2a).

The pigmentation intensity in the females of the 2 priEE-deleted strains (*w^1118^*; *e^Δ1028priEE^* and *w^1118^*; *e^Δ1029priEE^*), the control strain (*w^1118^*; +), and an *ebony* null mutant (*e^1^*) was compared. The % darkness at the specific positions of the thoracic center and the fourth abdominal tergite (A4) was measured (10 flies per strain) (Fig. 2b). The pigmentation scores were significantly different among the strains (thorax, *df* = 3, *χ*^2^ = 28.3, *P* < 10^−5^, Kruskal–Wallis rank sum test; abdomen, *df* = 3, *χ*^2^ = 26.0, *P* < 10^−5^, Kruskal–Wallis rank sum test). The thoraces of one of the 2 priEE-deleted strains, *w^1118^*; *e^Δ1028priEE^*, showed significantly but only slightly darker pigmentation than those of the control strain (Fig. 2c). The abdominal pigmentation in the 2 priEE-deleted strains was not significantly darker than that in the control strain (Fig. 2d). However, the pigmentation in the thorax and abdomen of the 2 priEE-deleted strains was markedly lighter than that in the thorax and abdomen of *e^1^* (Fig. 2, c and d). Although the pigmentation intensity is an indirect measurement of the transcription level of *ebony*, our results suggested that deletion had limited effects on the overall transcription level regulation. These results indicate that sequences driving expression at the epidermis resides outside the primary enhancer.

To estimate the expression levels of *ebony* by different means, the *mCherry* knocked-in strain, *w^1118^*; *e*::*mCherry*, and its priEE deleted strains, *w^1118^*; *e^Δ1017priEE^*::*mCherry* and *w^1118^*; *e^Δ1027priEE^*::*mCherry* were generated (Fig. 1c). The thoracic and abdominal pigmentation of *w^1118^*; *e^Δ1017priEE^*::*mCherry* and *w^1118^*; *e^Δ1027priEE^*::*mCherry* was largely consistent with the priEE-deleted strains without *mCherry* (*w^1118^*; *e^Δ1028priEE^ and w^1118^*; *e^Δ1029priEE^*) (Fig. 2 and Supplementary Fig. 6), which suggested that the catalytic function of Ebony in the pigmentation synthesis pathway is not disrupted upon fusion with mCherry.

The abdominal epidermis of the *mCherry* knocked-in strains were subjected to fluorescence confocal microscopy, which enabled us to visualize signal intensities from Ebony-mCherry (Fig. 3a). The anterior regions of A3–5 tergites were chosen as representative *ebony* expression domains with minimum effects of the bristles and stripes (Fig. 3a). The fluorescent intensities in these regions quantified from 10 females per strain were significantly different among the strains (*F*_4, 45_ = 14.6, *P* < 10^−6^, one-way ANOVA). The intensities of *w^1118^*; *e^Δ1017priEE^*::*mCherry* and *w^1118^*; *e^Δ1027priEE^*::*mCherry* were slightly higher than that in the control strain (Fig. 3b). Therefore, the priEE was shown to be not only dispensable for driving the transcription of *ebony* in the developing epidermis but may have slightly lower activity than the whole sequences outside itself.

Also, unexpectedly, the deletion of the priEE affected the pattern of pigmentation. In particular, the deletion of the priEE resulted in the loss of a dark pigmentation line along the dorsal midline of the abdominal tergites (Fig. 2a and Supplementary Fig. 6). This indicated that *ebony* expression is repressed in the midline area and that the priEE fragment is necessary for this suppression.

**Fig. 4:**
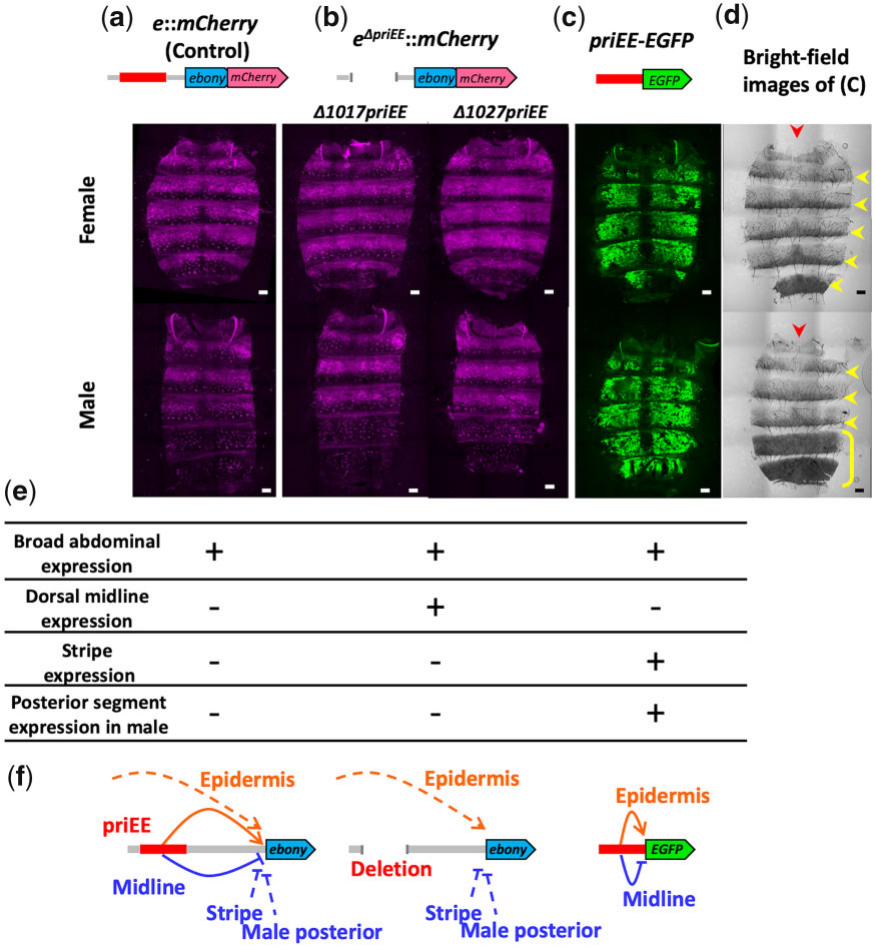
CREs regulate *ebony* expression in the developing epidermis. a) Confocal fluorescence images of the developing abdominal epidermis of *w^1118^*; *e*::*mCherry*. b) Confocal fluorescence images of the developing abdominal epidermis of *w^1118^*; *e^Δ1017priEE^*::*mCherry*, *w^1118^*; *e^Δ1027priEE^*::*mCherry*. c) Confocal fluorescence images of the developing abdominal epidermis of *priEE*-*EGFP* transformed to VK00033. d) Bright-field images of c). Images of females and males are shown in the upper and lower panels, respectively. The red arrowhead indicates the dorsal midline. The yellow arrowhead indicates a dark stripe on the posterior area of each tergite. The yellow bracket indicates male-specific dark pigmentation in the A5 and A6 tergites. Scale bars indicate 100 μm. e) Summary of the *ebony* expression sites for each strain determined from the fluorescence signals. f) The suggested model for the regulation of *ebony* expression in the abdomen. The solid lines indicate the effects of priEE, while the dotted lines indicate the effects of other CREs. The priEE fragment was also equipped to function as a midline silencer.

The expression sites of this gene in the abdomen were investigated by confocal microscopy of the *mCherry* knocked-in strains (Fig. 4, a and b). Endogenous *ebony* exhibited a broad epidermal expression pattern, and a suppressed expression at the posterior stripe region of each tergite (A1–6 of a female and A1–4 of a male) and tergite-wide suppression at A5 and A6 in males (Fig. 4, a, d, and e). These expression patterns were consistent with those reported in previous studies (Rebeiz and Williams 2017; Hughes *et al.* 2020). Notably, predicted from the lack of dark pigmentation, the expression of *e*::*mCherry* at the dorsal midline was not repressed in the priEE-deleted strains (Fig. 4, b and e).

The expression of *ebony* has been reported in other body regions (Hsouna *et al.* 2007; Suh and Jackson 2007; Rebeiz *et al.* 2009; Pérez *et al.* 2010) but the priEE has not been indicated to drive expression in tissues other than the developing epidermis (Rebeiz *et al.* 2009). As expected, the spatial expression patterns of *ebony* did not markedly change in other tissues upon deletion of the priEE (Supplementary Fig. 7).

The results indicated that the deleted priEE fragment contained a silencer element for the dorsal midline as well as an epidermal enhancer element. In order to confirm that the deleted priEE contains both elements, a GFP reporter assay was performed. The priEE fragment was fused to an enhanced GFP (*EGFP*) gene (*priEE*-*EGFP*) with a minimal Hsp70 promoter and transformed into 2 attP strains (VK00033 and VK00037). The confocal images of GFP from the homozygous *priEE*-*EGFP* transformed to a third chromosome landing site in VK00033 (Fig. 4, c and e) and a second chromosome landing site in VK00037 (Supplementary Fig. 8) indicated that the priEE autonomously drives the epidermal expression except at the flanking regions of the dorsal midline. However, the repression at the stripes and the male repression at the posterior segments were not observed (Fig. 4, c–e and Supplementary Fig. 8). Rebeiz *et al.* (2009) reported that the 0.7 kbp core element (included in the approximately 900-bp priEE) drove a similar expression pattern. The pattern clearly showed that a dorsal midline silencer is present in the priEE fragment and that it can silence the activity of the proximal enhancer element within the priEE fragment, which drives the broad expression of *ebony* in the developing abdominal epidermis.

To further narrow down the location of the silencer, the strains with partial deletions of the priEE were generated by injecting a mixture of pCFD5 vector that can produce 4 gRNAs (gRNA3–6) and pBFv-nosP-Cas9 vector into the embryos of *w^1118^*; *e*::*mCherry*. G1 individuals were screened for deletions by PCR-based genotyping (Supplementary Fig. 5). Out of 319 individuals screened, 14 were detected to have deletions. Among them, 4 individuals had breakpoints within the priEE. Two individuals had identical breakpoints described as *e^Δ1092priEE (5'_partial)^* and another 2 also had identical breakpoints described as *e^Δ498priEE (3'_partial)^* (Supplementary Fig. 3). Therefore, 1 homozygous strain each was established as in Supplementary Fig. 5.

**Fig. 5.**
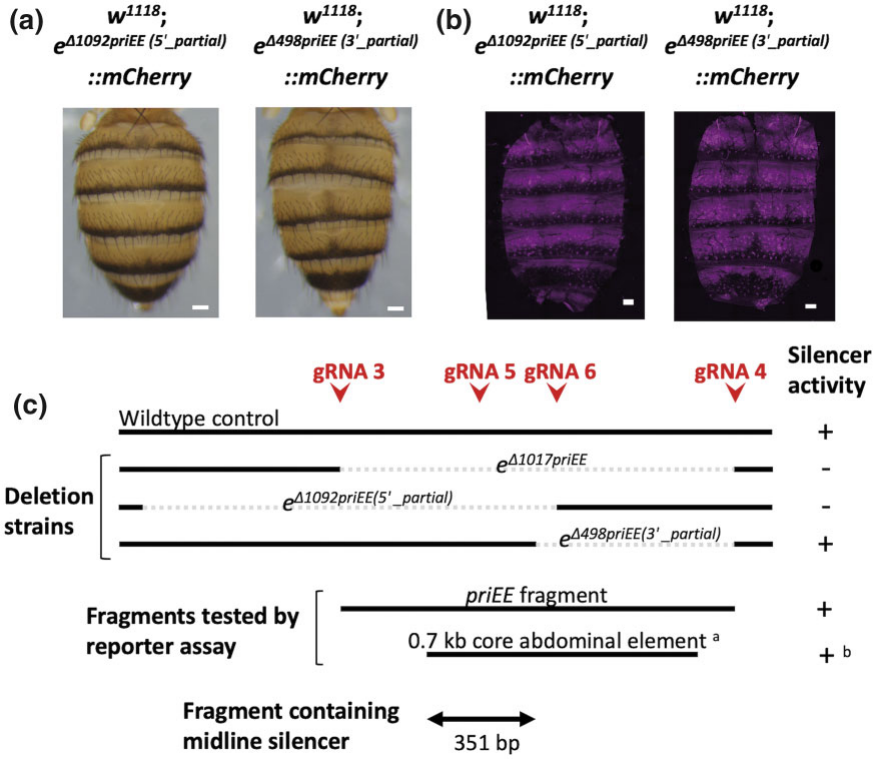
Location of the dorsal abdominal midline silencer within the priEE. a) The absence and presence of the dark pigmentation along the dorsal midline of the abdomen in *w^1118^*; *e^Δ1092priEE (5'_partial)^*::*mCherry* (left panel) and in *w^1118^*; *e^Δ498priEE (3'_partial)^*::*mCherry* (right panel), respectively. b) The absence and presence of Ebony-mCherry repression along the dorsal midline of the abdomen in *w^1118^*; *e^Δ1092priEE (5'_partial)^*::*mCherry* (left panel) and in *w^1118^*; *e^Δ498priEE (3'_partial)^*::*mCherry* (right panel), respectively. Scale bars indicate 100 μm. c) The summary of the breakpoints of the deletions and the tested fragments by reporter assays and the midline silencer activity detected in the corresponding strains. The red arrowheads indicate the positions of the gRNA target sites used to generate partial deletions of the priEE. Dotted lines indicate deleted positions. ^a^ and ^b^ are from Rebeiz *et al.* (2009). The double arrow indicates the position of the 351-bp fragment that includes the silencer.

The 2 strains with partially deleted priEE had different breakpoints; *w^1118^*; *e^Δ1092priEE (5'_partial)^*::*mCherry* and *w^1118^*; *e^Δ498priEE (3'_partial)^*::*mCherry* had deletions in the 5' and 3' portions of the priEE, respectively (Fig. 5c). The midline pigmentation status and Ebony-mCherry expression domains of these strains indicate that the silencer is removed in *w^1118^*; *e^Δ1092priEE (5'_partial)^*::*mCherry* but retained in *w^1118^*; *e^Δ498priEE (3'_partial)^*::*mCherry* (Fig. 5, a and b). The pattern indicated that the silencer resides in the 5' portion of the priEE. Together with the information that the midline expression of the *ebony* reporter driven by “0.7 kb abdominal core element” in Rebeiz *et al.* (2009) was repressed, the location of the silencer was indicated to be within the 351-bp overlapping region of this element and the non-deleted sequence of *w^1118^*; *e^Δ498priEE (3'_partial)^*::*mCherry* (Fig. 5c).

The fluorescent intensity (pixel value) in the abdominal epidermis of *w^1118^*; *e^Δ1092priEE (5'_partial)^*::*mCherry* was not significantly different from that of the control, but that of *w^1118^*; *e^Δ498priEE (3'_partial)^*::*mCherry* was slightly higher than that of the control (Fig. 3). These results indicate that the effect of deletions may slightly vary due to the differences in the breakpoint positions.

## Discussion

### Transcription-activating sequences other than the priEE reside in the *cis-*regulatory region of *ebony*

The removal of the endogenous priEE of *ebony* using the CRISPR-Cas9 system did not cause a drastic darkening as observed in the null mutant (*e*^1^), although a slight perturbation of the pigmentation intensities in the thoracic and abdominal segments was observed (Fig. 2 and Supplementary Fig. 6). A strong negative correlation between the darkness of body pigmentation and the expression level of *ebony* in the developing epidermis has been repeatedly detected in strains sampled from the natural populations of *D. melanogaster* (Pool and Aquadro 2007; Takahashi *et al.* 2007; Rebeiz *et al.* 2009; Takahashi and Takano-Shimizu 2011; Telonis-Scott *et al.* 2011; Miyagi *et al.* 2015). Thus, the dark pigmentation intensity of the cuticle could serve as a proxy indicator of local changes in the expression level of *ebony*, although compensation by other enzyme activities could influence the color. Therefore, the lack of a large increase in dark pigmentation in strains with the priEE deletion indicated that expression of the gene was not largely disrupted.

Another indirect but relevant comparison of the expression levels of *ebony* was conducted using the fluorescent intensity of mCherry fused to Ebony. The deletion of the whole priEE resulted in a slightly increased intensity of Ebony-mCherry (Fig. 3). These findings indicated that some endogenous sequences outside the priEE must have the ability to activate transcription of *ebony* in the developing epidermis, suggesting the presence of other enhancer element(s) in the surrounding genomic region. The complex arrangement of multiple CREs may be a reason for the scarcity of polymorphisms association with pigmentation intensity or gene expression level within or near the enhancer element across worldwide populations (Telonis-Scott and Hoffmann 2018).

The locations of other transcription-activating sequences have not been determined. The results of a previous reporter assay revealed that no fragments other than those including the priEE segment were detected within the approximately 10 kbp regulatory region that contains the 5' intergenic region and the first intron (Rebeiz *et al.* 2009). Therefore, any element that can drive epidermis expression is likely to be located elsewhere. However, unlike the recent reporter assay conducted with the *yellow* regulatory region (Kalay *et al.* 2019), many regions were tested using relatively large fragments (>2 kbp), which may contain cryptic enhancers that are repressed by surrounding sequences in their native genomic context. Therefore, the possibility of the presence of redundant enhancer elements within the approximately 10 kbp regulatory region cannot be ruled out. Some secondary enhancers are reported to be shadow enhancers that are more than 20 kbp away from the transcription start site (Hong *et al.* 2008). Thus, there is a need for extensive search to elucidate the detailed spatial arrangement of CREs. Nevertheless, the advantage of deleting an endogenous enhancer, a strategy employed in this study, is the rapid capturing of redundant enhancer activity in the native genomic context. Such knockout assays using endogenous genome editing may reveal more cases of redundant enhancer activities in the *Drosophila* genome as in the study conducting similar experiments on mouse developmental genes (Osterwalder *et al.* 2018). Moreover, this approach can compensate for some potential bias in reporter gene assays caused by the choice of promoter and the genomic location of the transgenes (Halfon 2019).

### Possible functions of redundant enhancers are to be investigated

The enhancer activities exhibited by sequences within and outside the priEE may be redundant in a sense that they both have abilities to drive transcription at the developing epidermis. However, how they coexist and function is not known. Redundant enhancer elements, which are often referred to as primary and shadow enhancers (Hong *et al.* 2008), have been suggested to confer robustness against environmental or genetic perturbations (Perry *et al.* 2009; Frankel *et al.* 2010; Pérez *et al.* 2010) or define sharp boundaries for gene expression (Perry *et al.* 2011; Bothma *et al.* 2015). The transcriptional activation of *ebony* by the sequences within and outside priEE appears to be largely overlapping but may not be completely redundant, considering the subtle changes in the pigmentation and Ebony-mCherry abundance upon deletion of the priEE (Fig. 2 and Supplementary Fig. 6). However, wide range of variations in the transcription level of this gene have been reported within and among *D. melanogaster* populations (Pool and Aquadro 2007; Takahashi *et al.* 2007; Rebeiz *et al.* 2009; Takahashi and Takano-Shimizu 2011; Telonis-Scott *et al.* 2011; Miyagi *et al.* 2015). Thus, maintaining a robust transcription level of this gene might not be essential. The functional significance of redundant enhancers in this gene requires further investigation.

### A silencer resides within an enhancer fragment

Spatially restricted suppression of focal gene transcription can be achieved by introducing a specific silencer that recruits repressive transcription factors (or repressors) expressed in the target cells. In contrast to enhancers, there is far limited information on the exact locations and features of silencers. This study demonstrated that the priEE fragment contained a silencer of *ebony* expression in the abdominal dorsal midline based on 2 experimental evidences. First, the repression of *ebony* expression at the dorsal midline was not observed when the priEE fragment was deleted (Fig. 4b). Second, experiments with priEE fragment fused to a reporter gene revealed a broad epidermal expression driven by autonomous enhancer activity and the repression of gene expression at the dorsal midline (Fig. 4c). Furthermore, we obtained 2 partial priEE deletions and observed the effects on the Ebony-mCherry expression. By comparing the results with that of a previous reporter assay (Rebeiz *et al.* 2009), we were able to narrow down the effective silencer fragment to 351 bp (Fig. 5). Thus, our study demonstrated that the combination of both deletion and reporter assays are effective in identifying silencer boundaries.

The *ebony* expression in the priEE-deleted strains implies that in the wild-type strain, when the silencer is intact, overall enhancer activity at the dorsal midline is blocked. Although not known, if multiple enhancers are driving expression of this gene simultaneously in the developing epidermis, they are likely to be repressed altogether by the presence of a single silencer. The results of the *priEE-EGFP* reporter assay demonstrated that the repressor bound to the silencer within the fragment interferes with the neighboring enhancer activity of priEE. Taken together, although the underlying mechanisms have not been elucidated, our results suggest that the silencer is sufficient to overcome overall enhancer activity that drives epidermal expression of this gene, possibly by interfering with the basal transcription machinery at the promoter site. A chromatin conformation analysis may be effective to identify the direct physical interaction between the silencer and the promoter.

Various models have been described to explain the functional categories of silencers and many silencers have been shown to act also as enhancers at different cellular contexts (Gray and Levine 1996; Courey and Jia 2001; Maston *et al.* 2006; Ogiyama *et al.* 2018; Gisselbrecht *et al.* 2020; Segert *et al.* 2021). The spatial configuration of the epidermis enhancer and the midline silencer within the priEE sequence is yet to be investigated. An identification of the actual binding sites of the positive- and negative-acting transcription factors should help clarifying the picture.

A schematic representation of the *cis*-regulatory transcriptional control of this gene is shown in Fig. 4f. As incorporated in the model, the repression of *ebony* in the dark stripes at the posterior regions of the abdominal tergites and the totally dark A5–6 tergites in males is not affected (Fig. 4, a–d). This is consistent with the results of a previous study, which showed that the locations of these silencers are not within the deleted fragment (Rebeiz *et al.* 2009). The authors revealed that the male silencer was located approximately 1.5 kbp upstream of the transcription start site, and the stripe silencer was located within the first intron. A similar approach to remove the putative silencer region can be effective for obtaining a comprehensive picture of the regulatory system of this gene.

At the molecular level, *omb*, *dpp*, and *wg*, are reported to be involved in the formation of sexually monomorphic pigmentation patterns in the abdomen of *D. melanogaster*, and *dpp*, which is expressed at the dorsal midline is essential for the formation of dark pigmentation along the midline (Kopp and Duncan 1997; Kopp *et al.* 1999; Wittkopp *et al.* 2003a). Additionally, *dpp* is known to activate the BMP signaling pathway, which regulates the transcription of numerous genes through a downstream transcription factor Mad (reviewed in Hamaratoglu *et al.* 2014). Kopp *et al.* (1999) showed that *Mad^12^* clones at or near the dorsal midline promoted the loss of dark pigmentation, which suggested that Dpp signaling contributes to pigmentation. Furthermore, an RNAi screening revealed that 48 transcription factors, including Mad, are involved in abdominal pigmentation (Rogers *et al.* 2014). Therefore, although not investigated in this study, there is a possibility that the repression of *ebony* by the silencer is regulated through the Dpp signaling pathway.

### Derepression of *ebony* is sufficient to diminish a taxonomic character

In the genus *Drosophila*, the pigmentation pattern of the abdominal midline is one of the traits used to classify the subgenus *Sophophora*, which includes *D. melanogaster*, and the subgenus *Drosophila*. With some exceptions, the pigmentation stripes on the abdominal tergites of the subgenus *Sophophora* are mostly connected or expanded anteriorly at the dorsal midline forming a distinct dark area along the midline as in *D. melanogaster* (Figs. 2a and 4d). In contrast, the stripes are narrowed or broken at the midline in most species of the subgenus *Drosophila* (Markow and O’Grady 2006). We have shown that the suppression of *ebony* by the abdominal midline silencer is at least necessary for the *Sophophora*-type midline to appear in *D. melanogaster*. The expression patterns of *pale*, *Ddc*, *ebony*, *tan*, and *yellow* in the developing abdominal epidermis of species belonging to subgenus *Sophophora* were previously examined using in situ hybridization (Hughes *et al.* 2020). Among the investigated genes, the suppression of *ebony* appears to be most pronounced in species with a typical dark dorsal midline.

A pair of sister species within the subgenus *Drosophila*, *D. americana*, and *D. novamexicana*, represents another case of distinct pigmentation patterns in the abdominal midline. *Drosophilaamericana* has a dark body color with uniformly dark abdominal tergites, whereas *D. novamexicana* exhibits a light pigmentation along the abdominal midline (Wittkopp *et al.* 2003b), which is a typical pattern of the subgenus. A recent study used reciprocal hemizygosity testing to demonstrate that the difference in abdominal midline pigmentation intensity between the 2 species was due to *ebony* (Lamb *et al.* 2020). The authors showed that *ebony* is required for the development of light pigmentation along the dorsal midline in wild-type *D. novamexicana*. It has not been demonstrated whether the interspecific differences of *ebony* reside in the *cis*-regulatory region or not. However, the study also suggests that *ebony* suppression might be a key factor for determining this taxonomically important trait.

In this study, the sequences outside the priEE did not exhibit any negative regulation at the dorsal abdominal midline region suggesting that a single silencer is suppressing the overall transcription driven by all the CREs of *ebony*. In contrast, a study of *yellow*, which is also expressed in the developing epidermis, revealed the presence of many short-range repressor binding sequences, which showed frequent evolutionary acquisition and loss among *D.melanogaster*, *D. pseudoobscura*, and *D. willistoni* (Kalay *et al.* 2019). Also, reporter assays examining the effect of the male-specific silencer of *ebony* in *D. auraria* and *D. serrata* observed a frequent loss of this silencer in these species (Johnson *et al.* 2015). The differences in silencer properties may be attributed to the evolutionary stability of the focal expression sites. The presence of a universal silencer may be prevalent in genes responsible for relatively stable taxonomic characters that delimit certain clades of species. Such silencers enable the redundant enhancer elements to fine-tune their regulation while maintaining robust transcription suppression in a spatially restricted manner.

These findings, together with the recently accumulating evidences of redundant CREs, suggest that the architectures of *cis*-regulatory regions are diverse and the possible evolutionary regimes may be more complex and variable than the general view of modularly restricted evolution of CREs.

## Data availability

Strains are available upon request. The authors affirm that all data necessary for confirming the conclusions of the article are present within the article, figures, and supplementary materials.

Supplementary material is available at *G3* online.

## Supplementary Material

jkac010_Supplementary_Figs

jkac010_Supplementary_Table1_Table2
