# Supplementary material for: The role of the epidermis enhancer element in positive and negative transcriptional regulation of ebony in Drosophila melanogaster
Source: G3 (Bethesda). 2022 Jan 13;12(3):jkac010. doi: 10.1093/g3journal/jkac010 (PMC8895987; doi:10.1093/g3journal/jkac010)
Supplement: jkac010_Supplementary_Figs [file jkac010_supplementary_figs.pdf]

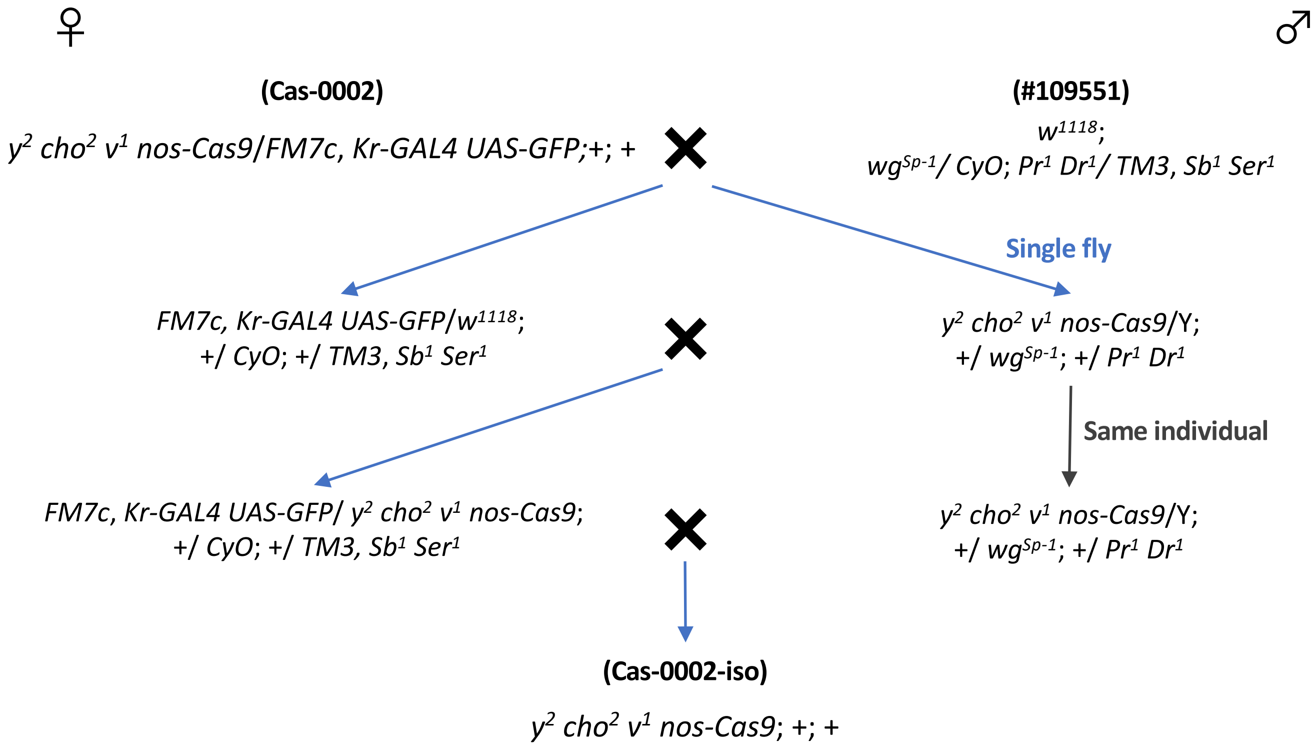

Supplementary Fig. 1 Scheme for extracting an isogenized chromosome

To control for the genetic background of the genome-edited flies, each chromosome was originated from a single chromosome. The isogenized strain was named Cas-0002-iso.

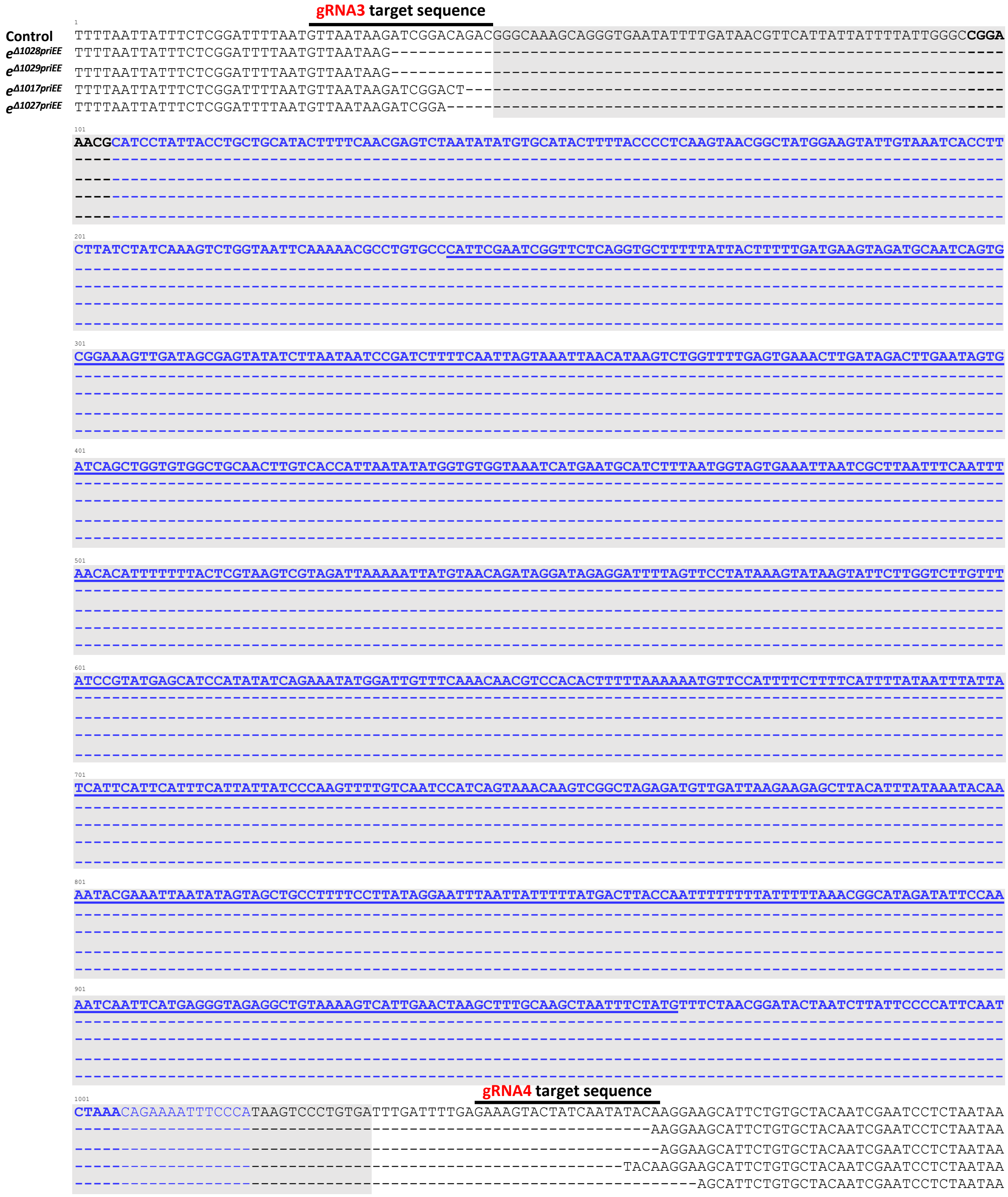

Supplementary Fig. 2 Alignment of sequences around the primary enhancer element (priEE) in the control and priEE knockout strains

The guide RNA (gRNA) target sequences are indicated above the sequence. Bases in bold indicate the 947-bp sequence of *e*_ECR0.9 from Takahashi and Takano-Shimizu (2011)(909-bp sequence in the control strain (Cas-0002-iso-derived w^{1118}; + strain)). Bases in blue indicate the 961-bp sequence of *e*_*core*_*cis* sequence from Miyagi *et al.* (2015) (915 bp in the control strain (Cas-0002-iso-derived w^{1118}; + strain) and the underlined bases indicate the "0.7 kb core abdominal element" from Rebeiz *et al.* (2009). Shaded bases indicate the sequence fragment (987 bp) used for the reporter assay in this study.

```
                                  -500
Control                           TGGAAACATAATCGATCATAACCAATCAAATTCGTGCATATACAGTCCTTCTTTCACCAAGCCAAATTCACGGCCTTATAAACCTGATAAGCATGTCTGT
eΔ1092priEE (5'_partial)          TGGAAACATAATCGATCATAACCG----------------------------------------------------------------------------
eΔ498priEE (3'_partial)           TGGAAACATAATCGATCATAACCAATCAAATTCGTGCATATACAGTCCTTCTTTCACCAAGCCAAATTCACGGCCTTATAAACCTGATAAGCATGTCTGT

-400
TACATTAGTTGTAGCTTTATCAGGTGCCCCAAACTATTAAAGTATTTCTTCCTTCAAAGTCACAGGTGCATTTGATCTTTAGATCAAGTATCTGAGCTCT
----------------------------------------------------------------------------------------------------
TACATTAGTTGTAGCTTTATCAGGTGCCCCAAACTATTAAAGTATTTCTTCCTTCAAAGTCACAGGTGCATTTGATCTTTAGATCAAGTATCTGAGCTCT

-300
CCATACATAACTACATATACATATAACTACATAATATACATAACCATTGAATGTTTCGCACCCGGACTAAAGCGATATTTTTTATAAGCGAATTCTGGTC
----------------------------------------------------------------------------------------------------
CCATACATAACTACATATACATATAACTACATAATATACATAACCATTGAATGTTTCGCACCCGGACTAAAGCGATATTTTTTATAAGCGAATTCTGGTC

-200
AAATGCCTAAGATAATGGTTAGTAATACGGATACAGGGAATTTAAATATCCGCAATCCACAAAAGGAATTCGTATTTTAAAAAAATCCCGAAAGTAGTAC
----------------------------------------------------------------------------------------------------
AAATGCCTAAGATAATGGTTAGTAATACGGATACAGGGAATTTAAATATCCGCAATCCACAAAAGGAATTCGTATTTTAAAAAAATCCCGAAAGTAGTAC

-100
TAACAGACAAATATTATTATTCCTCATATTAGCGCAAAATGAATAGTTATATTATTTATTATTTATCAAATTTATCTGCATGGTAATCATAAACCTCCAG
----------------------------------------------------------------------------------------------------
TAACAGACAAATATTATTATTCCTCATATTAGCGCAAAATGAATAGTTATATTATTTATTATTTATCAAATTTATCTGCATGGTAATCATAAACCTCCAG

                                            gRNA3 target sequence
1
TTTTAATTATTTCTCGGATTTTAATGTTAATAAGATCGGACAGACGGGCAAAGCAGGGTGAATATTTTGATAACGTTCATTATTATTTTTATTGGGCCGGA
----------------------------------------------------------------------------------------------------
TTTTAATTATTTCTCGGATTTTAATGTTAA--------------CGGGCAAAGCAGGGTGAATATTTTGATAACGTTCATTATTATTTTTATTGGGCCGGA

101
AACGCATCCTATTACCTGCTGCATACTTTTCAACGAGTCTAATATATGTGCATACTTTTACCCCTCAAGTAACGGCTATGGAAGTATTGTAAATCACCTT
----------------------------------------------------------------------------------------------------
AACGCATCCTATTACCTGCTGCATACTTTTCAACGAGTCTAATATATGTGCATACTTTTACCCCTCAAGTAACGGCTATGGAAGTATTGTAAATCACCTT

201
CTTATCTATCAAAGTCTGGTAATTCAAAAACGCCTGTGCCCATTCGAATCGGTTCTCAGGTGCTTTTTATTACTTTTTGATGAAGTAGATGCAATCAGTG
----------------------------------------------------------------------------------------------------
CTTATCTATCAAAGTCTGGTAATTCAAAAACGCCTGTGCCCATTCGAATCGGTTCTCAGGTGCTTTTTATTACTTTTTGATGAAGTAGATGCAATCAGTG

                                                                                          gRNA5 target sequence
301
CGGAAAGTTGATAGCGAGTATATCTTAATAATCCGATCTTTTCAATTAGTAAATTAACATAAGTCTGGTTTTGAGTGAAACTTGATAGACTTGAATAGTG
----------------------------------------------------------------------------------------------------
CGGAAAGTTGATAGCGAGTATATCTTAATAATCCGATCTTTTCAATTAGTAAATTAACATAAGTCTGGTTTTGAGTGAAACTTGATAGACTTGAATAGTG

401
ATCAGCTGGTGTGGCTGCAACTTGTCACCATTAATATATGGTGTGGTAAATCATGAATGCATCTTTAATGGTAGTGAAATTAATCGCTTAATTTCAATTT
----------------------------------------------------------------------------------------------------
ATCAGCTGGTGTGGCTGCAACTTGTCACCATTAATATATGGTGTGGTAAATCATGAATGCATCTTTAATGGTAGTGAAATTAATCGCTTAATTTCAATTT

501
AACACATTTTTTTACTCGTAAGTCGTAGATTAAAAATTATGTAACAGATAGGATAGAGGATTTTAGTTCCTATAAAGTATAAGTATTCTTGGTCTTGTTT
----------------------------------------------------------------------------------------------------
AACACATTTTTTTACTCGTAAGTCGTAGATTAAAAATTATGTAACAGATAGGATAGAGGATTTTA-----------------------------------

      gRNA6 target sequence
601
ATCCGTATGAGCATCCATATATCAGAAATATGGATTGTTTCAAACAACGTCCACACTTTTTAAAAAAATGTTCCATTTTCTTTTCATTTTATAATTTATTA
----------------ATATATCAGAAATATGGATTGTTTCAAACAACGTCCACACTTTTTAAAAAAATGTTCCATTTTCTTTTCATTTTATAATTTATTA
----------------------------------------------------------------------------------------------------

701
TCATTCATTCATTTCATTATTATCCCAAGTTTTGTCAATCCATCAGTAAACAAGTCGGCTAGAGATGTTGATTAAGAAGAGCTTACATTTATAAATACAA
TCATTCATTCATTTCATTATTATCCCAAGTTTTGTCAATCCATCAGTAAACAAGTCGGCTAGAGATGTTGATTAAGAAGAGCTTACATTTATAAATACAA
----------------------------------------------------------------------------------------------------

801
AATACGAAATTAATATAGTAGCTGCCTTTTCCTTATAGGAATTTAATTATTTTTATGACTTACCAATTTTTTTTATTTTTAAACGGCATAGATATTCCAA
AATACGAAATTAATATAGTAGCTGCCTTTTCCTTATAGGAATTTAATTATTTTTATGACTTACCAATTTTTTTTATTTTTAAACGGCATAGATATTCCAA
----------------------------------------------------------------------------------------------------

901
AATCAATTCATGAGGGTAGAGGCTGTAAAAGTCATTGAACTAAGCTTTGCAAGCTAATTTCTATGTTTCTAACGGATACTAATCTTATTCCCCATTCAAT
AATCAATTCATGAGGGTAGAGGCTGTAAAAGTCATTGAACTAAGCTTTGCAAGCTAATTTCTATGTTTCTAACGGATACTAATCTTATTCCCCATTCAAT
----------------------------------------------------------------------------------------------------

                                                gRNA4 target sequence
1001
CTAAACAGAAAATTTCCCATAAGTCCCTGTGATTTGATTTTGAGAAAGTACTATCAATATACAAGGAAGCATTCTGTGCTACAATCGAATCCTCTAATAA
CTAAACAGAAAATTTCCCATAAGTCCCTGTGATTTGATTTTGAGAAAGTACTAGGAAG-TACAAGGAAGCATTCTGTGCTACAATCGAATCCTCTAATAA
-----------------------------------------------------------------AGGAAGCATTCTGTGCTACAATCGAATCCTCTAATAA
```

Supplementary Fig. 3 Alignment of sequences around the primary enhancer element (priEE) in the control and the strains with partially deleted priEE

The guide RNA (gRNA) target sequences are indicated above the sequence. Bases in bold indicate the 947-bp sequence of *e*_ECR0.9 from Takahashi and Takano-Shimizu (2011)(909-bp sequence in the control strain (Cas-0002-iso-derived w^{1118}; + strain)). Bases in blue indicate the 961-bp sequence of *e*_*core*_*cis* sequence from Miyagi *et al.* (2015) (915 bp in the control strain (Cas-0002-iso-derived w^{1118}; + strain) and the underlined bases indicate the "0.7 kb core abdominal element" from Rebeiz *et al.* (2009). Shaded bases indicate the sequence fragment (987 bp) used for the reporter assay in this study. The red arrows indicate the first and the last nucleotides of the 351-bp abdominal midline silencer element.

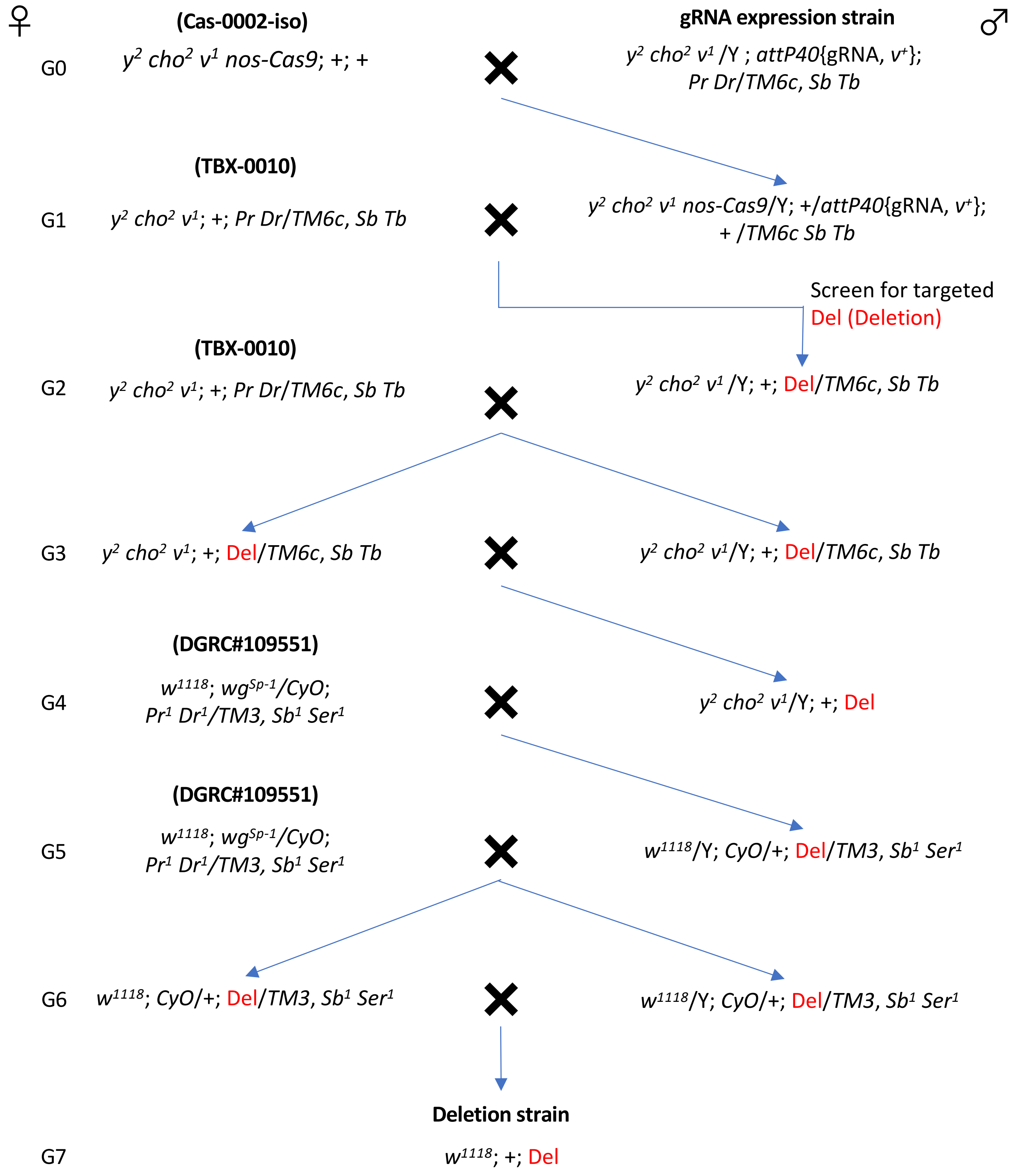

Supplementary Fig. 4 Scheme for generating primary enhancer element (priEE) deletion strains without y^2

CRISPR-Cas9-based genome editing was performed by crossing the Cas-0002-iso with the guide RNA (gRNA) expression strain (G0). The progenies from the G1 cross were screened for the presence of deletions. Homologous deletions were achieved by the crosses in G2 and G3. y^2 was removed by the crosses in G4 to G7 because it interferes with *ebony* in the pigment biosynthesis pathway. Deletion strains for mCherry fluorescence observation were established using the same scheme, except Cas-0002-iso_*e*::*mCherry* was used instead of Cas-0002-iso (G0). Control strains (w^{1118}; +; + and w^{1118}; +; *e*::*mCherry*) were established with the same crosses using TBX-0002 instead of the gRNA expression strain and using + [illegible]

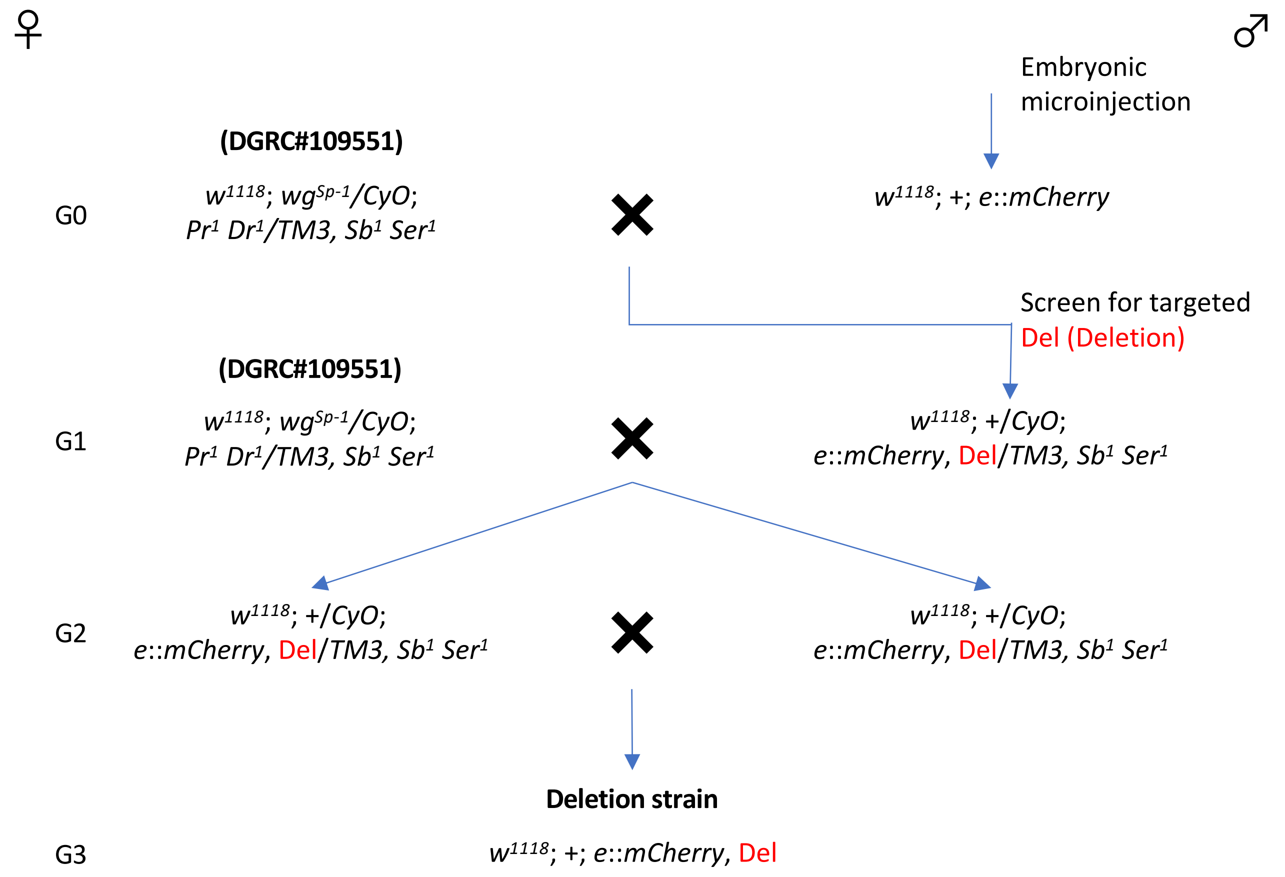

Supplementary Fig. 5 Scheme for generating strains with partially deleted primary enhancer element (priEE)

CRISPR-Cas9-based genome editing was performed by embryonic microinjection of gRNA3–6 cloned pCFD5 and pBFv-nosP-Cas9 vectors. The progenies from the G0 cross were screened for the presence of deletions. Homologous deletions were achieved by the crosses in G1 and G2.

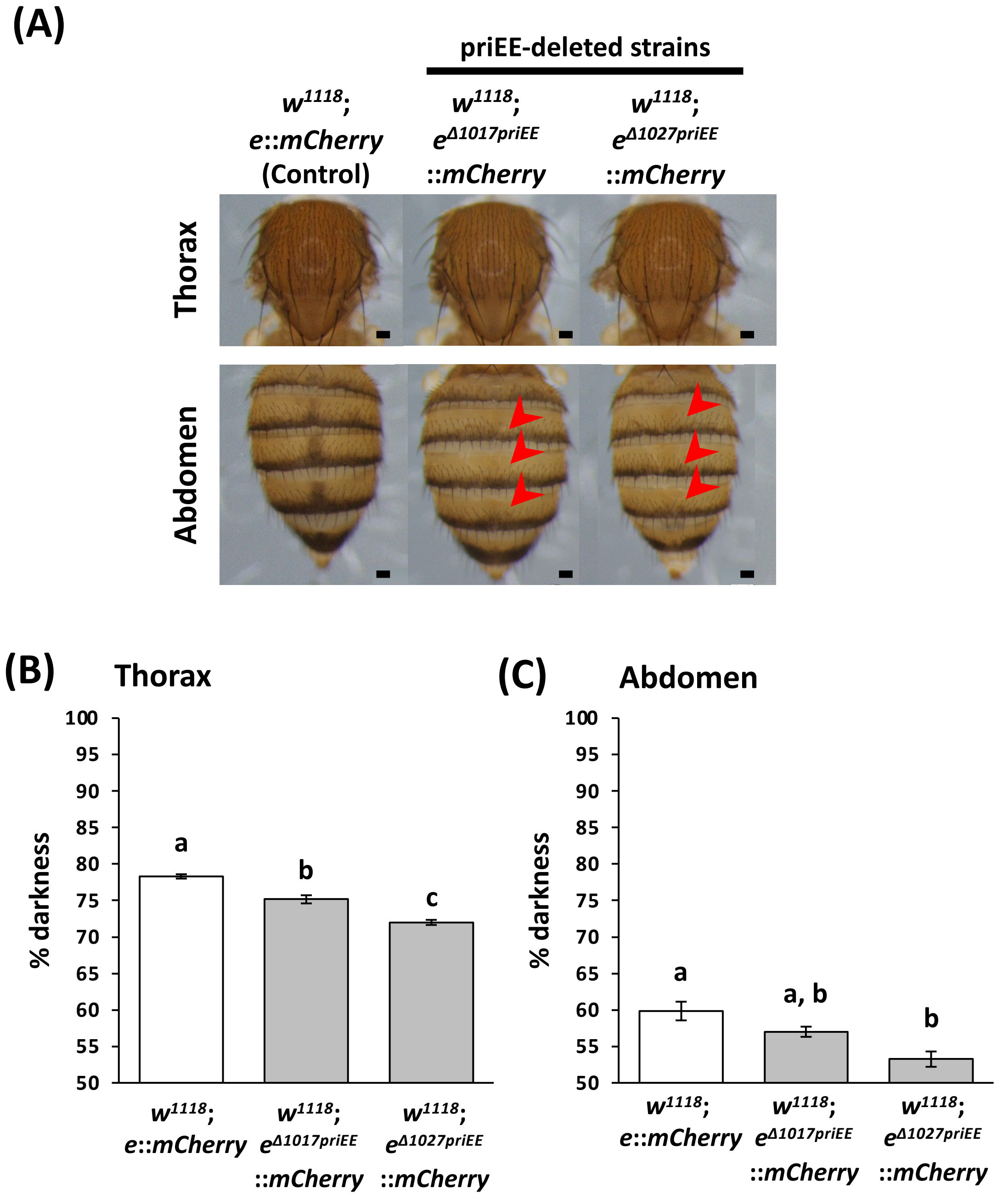

Supplementary Fig. 6 Effect of primary enhancer element (priEE) knockout on the intensity and patterns of pigmentation

(A) Images of the 5–7-day-old adult females. The red arrow indicates the area where the dark pigmentation in the dorsal midline is missing. (B) Percent (%) darkness values of the thorax. (C) Percent (%) darkness values of the A4 abdominal segment. Pigmentation of thorax and abdomen was quantified in the areas indicated by the yellow squares in the upper and lower panels, respectively, in Figure 2B. *N* = 10 for each strain. Different letters indicate significant differences between strains ($P < 0.05$; Kruskal-Wallis rank sum test followed by Dunn's test with Bonferroni correction). Scale bars indicate 100 μm and error bars denote standard error.

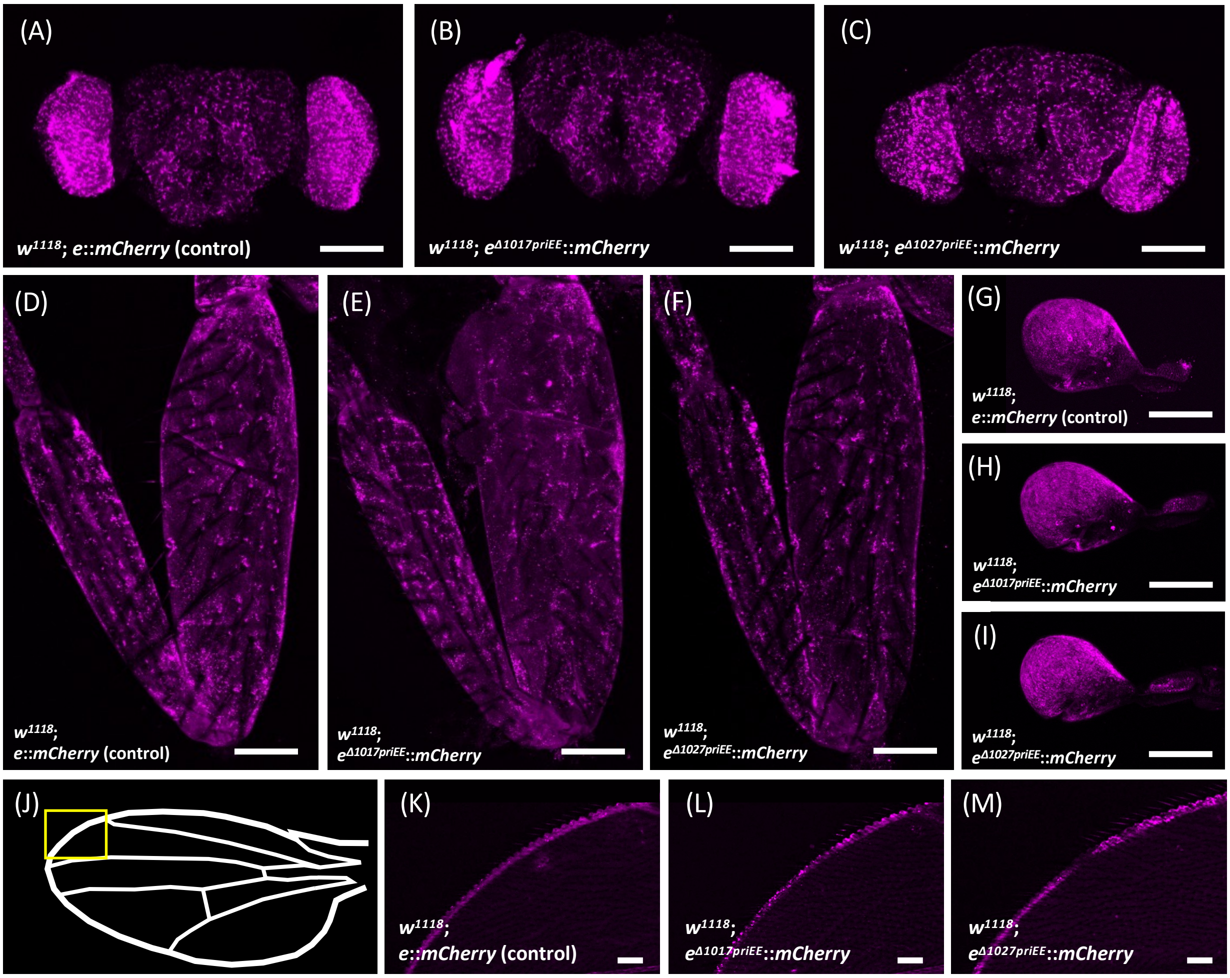

Supplementary Fig. 7 *ebony* expression in tissues other than developing epidermis

Confocal images of the mCherry-fused Ebony in the brain (A, B, and C), the front leg (D, E, and F), the haltere (G, H, and I), and the wing (K, L, and M) at the area indicated by the yellow square (J). Each tissue was dissected from the control, w^{1118}; *e*::*mCherry* (A, D, G, and K), and the priEE-deleted strains, w^{1118}; $e^{\Delta 1017priEE}$::*mCherry* (B, E, H, and L) and w^{1118}; $e^{\Delta 1027priEE}$::*mCherry* (C, F, I, and M). Scale bars indicate 100 μm.

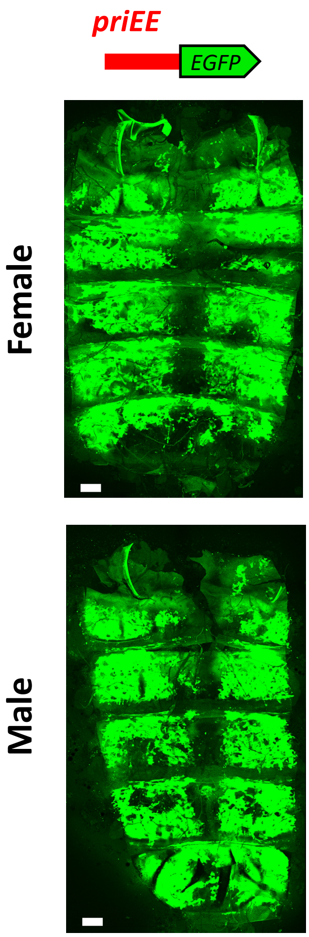

Supplementary Fig. 8 Reporter assay using the enhanced GFP-fused primary enhancer element (*priEE-EGFP*) construct in the developing abdominal epidermis

Confocal fluorescence images of the developing abdominal epidermis of *priEE-EGFP* transformed to VK00037. Images of females and males are shown in the upper and lower panels, respectively. Scale bars indicate 100 μm.
