# Supplementary material for: The role of the epidermis enhancer element in positive and negative transcriptional regulation of ebony in Drosophila melanogaster
Source: G3 (Bethesda). 2022 Jan 13;12(3):jkac010. doi: 10.1093/g3journal/jkac010 (PMC8895987; doi:10.1093/g3journal/jkac010)
Supplement: jkac010_Supplementary_Table1_Table2 [file jkac010_supplementary_table1_table2.pdf]

Supplementary Table 1 Raw pigmentation scores of the strains used in this study.

strain	Individual number	Thorax			Abdomen		
		Mode[a]	BG[b]	%darkness[c]	Mode[a]	BG[b]	%darkness[c]
w1118; + (control)	1	85	161	73.5	108	162	66.5
	2	89	162	72.4	109	162	66.2
	3	79	163	75.7	114	163	64.9
	4	83	164	74.6	110	162	65.9
	5	80	162	75.2	108	162	66.5
	6	78	161	75.7	108	163	66.7
	7	76	162	76.5	116	162	64.1
	8	81	162	74.9	120	163	63.0
	9	79	162	75.5	105	161	67.3
	10	76	161	76.3	106	162	67.2
w1118; $e^{\Delta 1028priEE}$	1	66	160	79.3	119	160	62.7
	2	63	161	80.4	120	159	62.1
	3	67	159	78.8	131	160	58.9
	4	66	160	79.3	133	160	58.3
	5	60	161	81.3	121	161	62.3
	6	67	160	79.0	124	163	61.8
	7	71	161	77.9	133	162	58.8
	8	64	161	80.0	111	163	65.8
	9	67	160	79.0	122	162	62.2
	10	63	161	80.4	119	161	62.9
w1118; $e^{\Delta 1029priEE}$	1	66	159	79.2	117	160	63.3
	2	68	160	78.7	112	159	64.6
	3	66	160	79.3	108	163	66.7
	4	64	161	80.0	107	160	66.4
	5	69	160	78.4	117	160	63.3
	6	68	160	78.7	109	160	65.8
	7	64	160	79.9	119	161	62.9
	8	71	161	77.9	116	161	63.8
	9	65	161	79.7	133	160	58.3
	10	68	161	78.8	117	163	64.0
e^1	1	25	159	92.1	88	159	72.2
	2	24	161	92.5	86	159	72.8
	3	23	160	92.8	76	158	75.9
	4	22	161	93.1	70	159	77.9
	5	23	160	92.8	69	159	78.2
	6	23	161	92.8	70	159	77.9
	7	25	158	92.1	73	159	77.0
	8	23	159	92.7	74	160	76.8
	9	21	160	93.4	76	161	76.3
	10	24	160	92.5	76	159	76.0

[a] Mode: the mode of grayscale brightness values (0-255) of the quantified area (N = 10 females for each strain)

[b] BG: the mode of grayscale brightness values (0-255) of the background area (N = 10 females for each strain)

[c] % darkness was calculated from Mode corrected by the reference grayscale (128) of the background.

Supplementary Table 2 Raw Ebony-mCherry fluorescent intensity values of the strains used in this study

strain	Individual number	Mode A3 pixel value[a]	Mode A4 pixel value[a]	Mode A5 pixel value[a]	Average pixel value	Mode (background) pixel value	mCherry signal intensity[b]
w1118; e::mCherry (control)	1	23	24	25	24.00	3	21.00
	2	27	25	21	24.33	3	21.33
	3	20	17	16	17.67	3	14.67
	4	21	23	21	21.67	3	18.67
	5	26	25	22	24.33	3	21.33
	6	23	28	26	25.67	3	22.67
	7	25	26	26	25.67	3	22.67
	8	20	24	23	22.33	3	19.33
	9	23	24	21	22.67	3	19.67
	10	26	24	22	24.00	3	21.00
w1118; $e^{\Delta 1017priEE}$ ::mCherry	1	29	33	33	31.67	3	28.67
	2	31	30	34	31.67	3	28.67
	3	34	30	32	32.00	3	29.00
	4	28	25	30	27.67	3	24.67
	5	31	32	36	33.00	3	30.00
	6	24	27	28	26.33	3	23.33
	7	23	26	27	25.33	3	22.33
	8	22	29	25	25.33	3	22.33
	9	30	31	31	30.67	3	27.67
	10	31	31	33	31.67	3	28.67
w1118; $e^{\Delta 1027priEE}$ ::mCherry	1	29	33	29	30.33	3	27.33
	2	35	37	33	35.00	3	32.00
	3	34	35	34	34.33	3	31.33
	4	29	29	30	29.33	3	26.33
	5	32	32	34	32.67	3	29.67
	6	34	34	34	34.00	5	29.00
	7	29	32	34	31.67	3	28.67
	8	30	33	31	31.33	3	28.33
	9	29	32	35	32.00	3	29.00
	10	30	31	31	30.67	3	27.67
w1118; $e^{\Delta 1092priEE(5'\_partial)}$::mCherry	1	25	28	31	28.00	3	25.00
	2	23	24	23	23.33	3	20.33
	3	30	27	29	28.67	3	25.67
	4	23	22	24	23.00	3	20.00
	5	24	25	25	24.67	3	21.67
	6	23	28	31	27.33	3	24.33
	7	23	25	28	25.33	3	22.33
	8	26	29	31	28.67	3	25.67
	9	29	29	31	29.67	3	26.67
	10	26	28	30	28.00	3	25.00
w1118; $e^{\Delta 498priEE(3'\_partial)}$::mCherry	1	27	27	31	28.33	3	25.33
	2	27	28	31	28.67	3	25.67
	3	28	25	25	26.00	3	23.00
	4	21	24	25	23.33	3	20.33
	5	24	24	25	24.33	3	21.33
	6	32	31	34	32.33	3	29.33
	7	32	34	36	34.00	3	31.00
	8	30	35	34	33.00	3	30.00
	9	29	33	34	32.00	3	29.00
	10	29	31	33	31.00	3	28.00

[a] Manually chosen areas for measurements in A3-5 abdominal tergites are shown in Figure 3A. N = 10 females for each strain.

[b] mCherry signal intensity is calculated as "average pixel value" - "background pixel value".
